# Tunable Particle Separation in a Straight Microchannel via Symmetrical Viscoelastic Sheath Flows

**DOI:** 10.3390/bios16050273

**Published:** 2026-05-08

**Authors:** Tianyuan Zhou, Qi Cui, Guizhong Tian, Jing Xia, Ping Liu, Yoichiroh Hosokawa, Yaxiaer Yalikun, Pan Wang, Shilun Feng, Tianlong Zhang

**Affiliations:** 1 College of Mechanical Engineering, Jiangsu University of Science and Technology, Zhenjiang 212100, China; tianyuanzhou@stu.just.edu.cn (T.Z.);; 2 School of Mechanical and Electrical Engineering, Suqian University, Suqian 223800, China; 3 Medilux Research Center, Nara Institute of Science and Technology, Nara 630-0192, Japan; 4 State Key Laboratory of Transducer Technology, Shanghai Institute of Microsystem and Information Technology, Chinese Academy of Sciences, Shanghai 200050, China

**Keywords:** microfluidics, tunable separation, viscoelastic flows, symmetrical sheath, downstream processing

## Abstract

In this study, we present a novel microfluidic platform for tunable size-based particle separation within a straight microchannel using symmetrical viscoelastic sheath flows. The device incorporates two pairs of symmetrical microchannels for sheath fluid injection: the first pair facilitates particle focusing and separation, while the second pair enables dynamic regulation of the separation distance between particle streams. Experimental results demonstrate that a 50 ppm polyethylene oxide (PEO) solution focuses 1 μm polystyrene particles toward the channel centerline via elastic forces, whereas 5 μm particles migrate toward the channel sidewalls under dominant inertial forces, effectively overcoming the elastic effects. The interplay between inertial and elastic forces thus achieves size-dependent particle separation. Furthermore, by adjusting the flow rate of the PEO sheath in the second pair of microchannels, the separation distance between the two particle populations can be modulated in real time. Higher PEO concentrations (500 and 1000 ppm) exhibit enhanced capabilities to deflect particle flow streams. By contrast, the lower PEO concentrations like 50, 100 and 200 ppm are more versatile in adjusting the separation distance. The biological applicability of this platform is further demonstrated through the tunable separation of *Escherichia coli* (*E. coli*) and *Chlorella vulgaris* (*C. vulgaris*). This microfluidic device demonstrates significant potential for downstream particle processing applications, including real-time particle detection and targeted drug delivery.

## 1. Introduction

Viscoelastic microfluidics is a passive microfluidic strategy that exploits the nonlinear elastic properties of polymer-based viscoelastic fluids to manipulate particles or cells within microchannels without the need for external fields [1]. In a rectangular straight channel, the positions of suspended particles are equilibrated by the balance between elastic forces and inertial lift forces [2]. The elastic force, arising from the first normal stress difference generated by stretched polymer chains under shear flow, drives particles toward the channel centerline. In contrast, the inertial lift force, comprising shear-gradient and wall-induced components, induces lateral migration of particles toward equilibrium positions near the channel sidewalls. A wide range of water-soluble and biocompatible polymers, such as deoxyribonucleic acid (DNA) [3], xanthan gum (XG) [4], polyethylene oxide (PEO) [5] and polyvinylpyrrolidone (PVP) [6], are commonly employed to formulate viscoelastic fluids for experimental studies. One of the notable advantages of viscoelastic microfluidics lies in its tunability, as the magnitude of elastic forces can be readily controlled by adjusting the polymer concentration or flow rate. Owing to the benefits, viscoelastic microfluidics has gained significant attention in recent years and has been extensively applied to particle focusing [7], mixing [8], and size-based separation [9].

Various microchannel geometries have been developed to achieve size-dependent particle separation in viscoelastic microfluidics, enabling the label-free isolation of biological targets ranging from bacteria to blood cells and cancer cells [10]. Among these, straight rectangular microchannels represent one of the simplest and most widely adopted configurations, where elastic lift forces generated by viscoelastic fluids drive particles toward distinct equilibrium positions based on size, facilitating the isolation of biological targets such as breast cancer cells [11], *Escherichia coli* (*E. coli*) [12], platelets [6], and white blood cells [13] from complex sample mixtures. In addition to straight channels, several studies have explored curved or contraction–expansion geometries that induce secondary flow fields such as Dean vortices. In these designs, the combined action of Dean drag forces, elastic forces, and inertial lift forces enables efficient size- or deformability-based separation of cells, including *Nostoc* [14], *Enterococcus faecalis* [15], and mesenchymal stem cells [16]. Furthermore, sheath-assisted viscoelastic microfluidic platforms have been introduced to enhance separation precision by confining sample streams and exploiting interfacial effects between fluids of differing viscoelastic properties. Such configurations enable the synergistic utilization of inertial, elastic, and interfacial forces, thereby achieving effective separation of platelets [17], leukemia cells [18], yeast cells [19], *E. coli* [20], breast cancer cells [21], and extracellular vesicles [22,23].

Although viscoelastic microfluidics is versatile for particle separation, it lacks the capability to regulate the separation distance between particle streams [24,25]. On the other hand, other microfluidic techniques have achieved the regulation. For instance, Sai et al. developed an adjustable pinched flow fractionation (PFF) method that employs microvalves to regulate the flow distribution to each outlet, enabling precise collection of target particles from the desired outlet [26]. Jung et al. proposed an inertial microfluidic device capable of modulating the focusing positions of particles and altering fluid flow characteristics by adjusting the three-dimensional geometry of the microchannel, particularly its curvature radius [27]. Zeming et al. introduced a real-time control strategy for nanoparticle separation based on deterministic lateral displacement (DLD), which allows for the dynamic manipulation of separation by adjusting the ion concentration in the fluid medium [28]. Nevertheless, approaches for passive real-time separation control remain scarce, with the majority of current regulation strategies relying on active separation techniques such as dielectrophoresis [29], acoustofluidics [30,31,32], and optical tweezers [33]. These active methods, however, often require coupling with image analysis or programming technologies and depend on external force fields, resulting in increased system complexity.

In this study, two pairs of symmetric viscoelastic sheath flows were employed to achieve tunable separation of 1 μm and 5 μm particles. The first pair of sheath flows, consisting of a 50 ppm PEO solution, confined the particle suspension (also prepared in 50 ppm PEO) into a narrow stream at the channel center. Under these conditions, the 1 μm particles were focused along the centerline due to dominant elastic forces, whereas the 5 μm particles migrated toward the channel sidewalls under the influence of shear-gradient lift forces. Following the initial separation, the second pair of PEO sheath solutions, with concentrations of 50 ppm, 100 ppm, and 200 ppm, enabled modulation of the lateral separation distance between the 1 μm and 5 μm particle streams. Furthermore, the developed microfluidic platform successfully achieved tunable, size-dependent separation of *E. coli* and microalgae. This device exhibits strong potential for integration into downstream processing workflows, particularly for real-time particle detection and targeted drug delivery applications.

## 2. Materials and Methods

### 2.1. Channel Design and Device Fabrication

**Figure 1:**
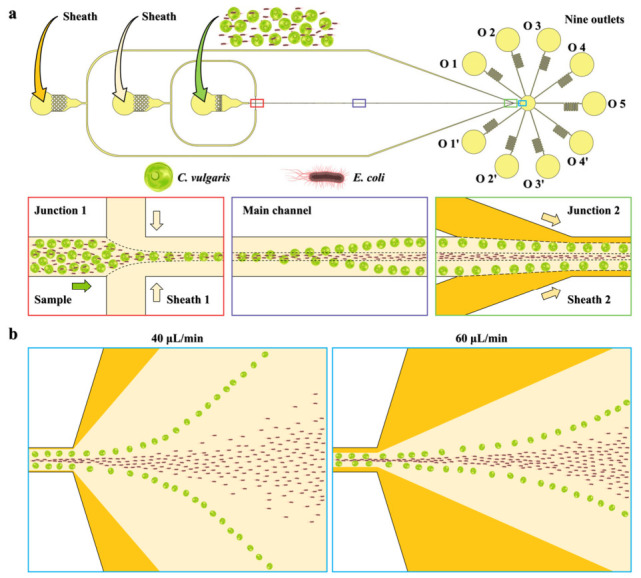
Schematic illustration of the tunable particle separation by size via symmetrical viscoelastic sheath fluids in a straight microchannel. (**a**) Channel design and operational principle. (**b**) Separation performances under different experimental scenarios. *C. vulgaris* and *E. coli* denote *Chlorella vulgaris* and *Escherichia coli*, respectively.

A microchannel comprising three inlets, a 2 cm long and 20 μm wide straight rectangular microchannel, two symmetric sheath flow structures, an expansion region, and nine outlets was designed (Figure 1). The microchannel had a uniform height of 50 μm. The microchannel mold was fabricated by patterning SU-8 2050 (MicroChem Corp., Westborough, MA, USA) onto a silicon wafer (MCL Electronic Materials Co., Ltd., Luoyang, Henan, China) using a direct-write maskless lithography system (MicroWriter ML3, Durham Magneto Optics Ltd, Sedgefield, County Durham, UK). Polydimethylsiloxane (PDMS, Sylgard 184, Dow Corning, Midland, MI, USA) base and curing agent were mixed at a weight ratio of 10:1, followed by vacuum degassing for 60 min. The PDMS mixture was then poured onto the mold and cured in an oven at 90 °C for over 2 h. After peeling the PDMS from the mold, inlet and outlet holes were punched. Before bonding, any debris on the PDMS and glass substrate surfaces was removed using blue polyethylene protective film adhesive. Finally, after treatment with a plasma cleaner (PLUTO-T, Shanghai Peiyuan Instrument Equipment Co., Ltd., Shanghai, China) at 70 W for 50 s, the PDMS layer was bonded to a coverslip (MICROSCOPE SLIDES, Citotest Labware Manufacturing Co., Ltd., Nantong, Jiangsu, China) and placed in an oven at 90 °C overnight to enhance the bonding strength.

### 2.2. PEO Solutions

In this study, polyethylene oxide (PEO) powder with a molecular weight of 600 kDa was employed to prepare viscoelastic solutions. Different amounts of PEO were dissolved in phosphate-buffered saline (PBS) within 50 mL centrifuge tubes (BY-3073, Shanghai Bingyu Fluid Technology Co., Ltd., Shanghai, China). The solutions were subsequently placed on an orbital shaker (HY-2H, Shanghai Xiniu Leibo Instrument Co., Ltd., Shanghai, China) and oscillated at 150 revolutions per minute (rpm) for over 24 h at room temperature to ensure complete dissolution. PEO solutions with concentrations of 20 ppm, 50 ppm, 100 ppm, 200 ppm, 500 ppm, and 1000 ppm were prepared accordingly for experimental use.

### 2.3. Particle Preparation

Polystyrene particles with diameters of 1 ± 0.1 µm and 5 ± 0.5 µm (density: 1.06 g/cm^3^, BaseLine, Tianjin BaseLine ChromTech Research Centre, Tianjin, China) were purchased and stored at 4 °C prior to use. In this study, the 1 µm and 5 µm particles were diluted in either PBS or PEO solutions to final concentrations of 7.5 × 10^7^ particles/mL and 3.6 × 10^6^ particles/mL, respectively. To prevent particle aggregation and adhesion to the microchannel walls, Tween 20 (Roche Diagnostics GmbH, Mannheim, Germany) was added to all suspensions at a concentration of 0.5%. Before introducing the particle suspension into the microchannel, the sample was thoroughly mixed using a pipette to ensure a homogeneous particle distribution.

### 2.4. Preparation of Microalgae and Escherichia coli (E. coli)

*E. coli* (strain number: HZB508667, Wuhan Huizao Biotechnology Co., Ltd., Wuhan, Hubei, China) has a size range of 0.5–1 μm in diameter and 1–4 μm in length along the long axis. The bacteria were cultured overnight at 37 °C on Tryptic Soy Agar (TSA) supplemented with 50 mcg/mL ampicillin. Subsequently, the bacterial cells were fixed with 70% ethanol at 20 °C for 1 to 3 h. After fixation, they were washed twice with phosphate-buffered saline (PBS) to remove the ethanol. Finally, the cells were resuspended in PBS and stored at 4 °C in a refrigerator for experimental use. *Chlorella vulgaris* (*C. vulgaris*) (strain number: GY-D27, Shanghai Guangyu Biotechnology Co., Ltd., Shanghai, China) has a size range of 3–8 μm. It was cultured in BG11 medium under alternating 12-h light (1000 lux) and 12-h dark cycles at 23 °C and prepared for subsequent use. In this study, *E. coli* and *C. vulgaris* were diluted with PBS and PEO solutions, respectively, to the desired final concentrations. The concentration of *E. coli* was 2.2 × 10^8^ cells/mL, and that of *C. vulgaris* was 6.8 × 10^7^ cells/mL. To prevent aggregation of the biological particles and their adhesion to the microchannel walls, 0.5% Tween 20 was added to all suspensions as a surfactant. Before introducing the particle suspension into the microchannel, the samples were thoroughly mixed using a pipette to ensure uniform particle distribution. Biological particles were recorded as they flowed toward the channel outlet to accurately evaluate collection performance, thereby circumventing the difficulties associated with regulating the outlet pressure.

### 2.5. Setup of Injection and Observation Systems

The microfluidic device was placed under an optical microscope (HRX-01, Hirox Co., Ltd., Tokyo, Japan). Sheath fluid and sample solutions were infused into the inlets of the microchannel via three syringe pumps (LSP02-1Y, Baoding Rongbai Precision Pump Manufacturing Co., Ltd., Baoding, China). The positions of particles and cells flowing through the microchannel were recorded using screen capture software (EVCapture, version 5.5.2, Hunan Ieway Information Technology Co., Ltd., Changsha, China). Experimental images were post-processed using ImageJ (version 1.54p, National Institutes of Health, Bethesda, MD, USA).

## 3. Working Mechanism

In straight microchannels, the migration of particles in viscoelastic microfluidics is governed by the combined influence of the net inertial lift force ($F_{L}$) and the elastic force ($F_{E}$) [2]. $F_{L}$ itself consists of the shear-gradient lift force ($F_{LS}$), which drives neutrally buoyant particles toward the nearby channel wall, and the wall-induced lift force ($F_{LW}$), which pushes particles from the wall toward the channel center [19]. Here, $F_{L}=F_{LS}+F_{LW}=\rho_{f}V_{m}d^{4}D_{h}^{-2}f_{L}$, where $\rho_{f}$ is the fluid density, $V_{m}$ is the mean flow velocity, and d, $D_{h}$, and $f_{L}$ represent the particle diameter, channel hydraulic diameter, and inertial lift coefficient, respectively [34]. However, for particles below several micrometers in size, $F_{L}$ is rendered negligible, as the inertial lift force diminishes sharply with reducing particle diameter.

In viscoelastic fluids, the reorientation and alignment of macromolecules along the flow direction give rise to normal stresses. Driven by the first normal stress difference $N_{1}$, the elastic force $F_{E}$ pushes particles toward the centerline, which corresponds to the region of the lowest shear rate. For particles smaller than a few micrometers, $F_{E}=C_{el}d^{3}\nabla N_{1}$ (where $C_{el}$ is the elastic lift coefficient) becomes significant [34]. Here, the first normal stress $N_{1}$ is defined as the difference between the streamwise normal stress $\sigma_{xx}$ and the transverse normal stress $\sigma_{yy}$, with the coordinate system set as follows: x is along the downstream flow direction, and y is perpendicular to the x-direction [1]. Therefore, the cross-sectional migration behavior of particles may be influenced by variations in several factors, including viscoelastic medium concentration, particle size, channel dimensions, and fluid velocity.

In this study, particle suspensions prepared in a 50 ppm PEO solution were hydrodynamically pinched into a narrow stream at the channel center by the first pair of 50 ppm PEO sheath flows, primarily driven by hydrodynamic drag forces [4,10] (Figure 1a). Once confined to the central streamline, 1 μm particles remained focused near the channel center due to the dominance of elastic forces acting on smaller particles in viscoelastic fluids [35,36]. In contrast, 5 μm particles experienced stronger shear-gradient lift forces owing to their larger size, causing their lateral migration away from the center toward the channel sidewalls. To characterize the competition between inertial and elastic forces, three dimensionless numbers were employed in this study: the Reynolds number ($Re=\rho V_{m}D_{h}/\mu$, where μ is the fluid viscosity), representing the ratio of inertial to viscous forces; the Weissenberg number ($Wi=\lambda \dot{\gamma }$, where λ is the fluid relaxation time and $\dot{\gamma }$ is the shear rate), quantifying the ratio of elastic to viscous forces; and the elasticity number ($El=W_{i}/Re$), indicating the importance of elastic forces relative to inertial forces [19,36]. Accordingly, the *Re*, *Wi*, and *El* vary with changes in flow velocity, and PEO concentration was provided (see Supplementary Table S1). In the downstream region near the end of the main channel, the lateral positions of the 5 μm particles were further modulated by the second pair of PEO sheath flows, enabling tunable separation performance (Figure 1b).

## 4. Results and Discussion

### 4.1. Effect of Total Flow Rate (TFR) on Separation Performance

**Figure 2:**
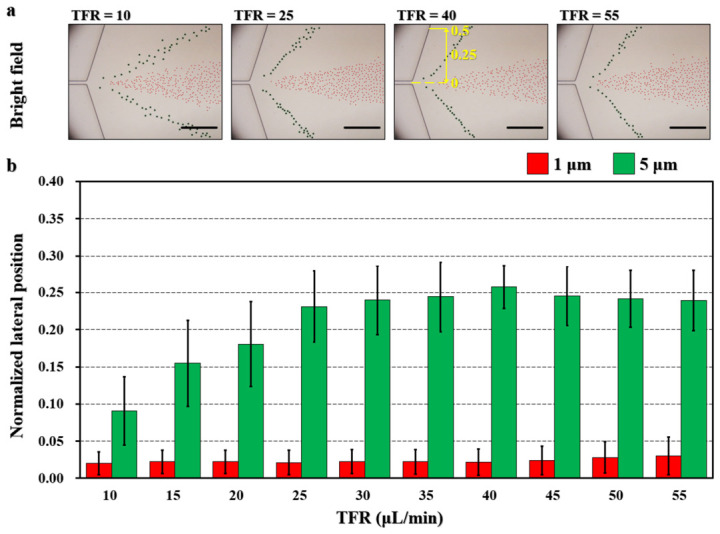
Effect of total flow rate (TFR) on the separation of 1 μm (red) and 5 μm (green) particles. (**a**) Superimposed bright-field images showing the particle distribution at the expansion of the microchannel. Scale bar is 200 μm. (**b**) Normalized lateral positions of 1 μm and 5 μm particles. TFRs were 10, 15, 20, 25, 30, 35, 40, 45, 50, and 55 μL/min. The flow rate of the outside PBS sheath was 5 μL/min. The flow rate ratio (FRR) between the middle sheath flow and the sample flow was fixed at 4:1. *N* = 200 for each condition.

To evaluate the effect of TFR on the separation efficiency of the microfluidic device, the flow rate ratio (FRR) between the middle sheath flows and the sample stream was fixed at 4:1. The particle suspensions were prepared in a 50 ppm PEO solution. The 50 ppm PEO solution was also used as the fluid for the inner sheath flows. To prevent backflow of the sample solution into the second sheath flow microchannel, phosphate-buffered saline (PBS), a Newtonian fluid, was introduced into the inlets of the second sheath pair at a constant flow rate of 5 μL/min. A series of ten TFRs, ranging from 10 to 55 μL/min in increments of 5 μL/min (including both sample and two pairs of sheath flows), was investigated. In the provided images, 1 μm and 5 μm particles are indicated with red and green circles, respectively, for clarity. The lateral positions of particles in the downstream expansion region were recorded, analyzed, and compared to assess separation performance (Figure 2). The separation performance of the microfluidic platform was evaluated by analyzing the normalized particle positions across the lateral direction of the expansion region (2.02 cm downstream of the channel inlet), as shown in Figure 2a. The straight yellow line indicates the normalized positions of the particle distribution across the channel direction. Data are presented as mean ± standard deviation.

Superimposed bright-field images indicated that under all TFR conditions, 1 μm particles flowed along the channel centerline, while the lateral displacement of 5 μm particles exhibited TFR-dependent behavior (Figure 2a). Normalized lateral positions of 5 μm particles increased from 0.09 at 10 μL/min to 0.26 at 40 μL/min (Figure 2b). A video demonstrating the separation performance at a flow rate of 40 μL/min in the expansion region was provided (Video S1). However, further increases in TFR failed to maintain this increasing tendency because of the inertia of the flowing 5 μm particles [37]. At a TFR of 40 μL/min, 5 μm particles were positioned near the channel sidewalls under the coupled effects of inertial and elastic forces. In contrast, 1 μm particles were focused into a narrow stream, as elastic forces dominated over inertial forces. A simulation on the flow rate distribution is provided to promote the understanding (Figure S1). The differences in the average normalized lateral positions between 1 μm and 5 μm particles were 0.07 (10 μL/min), 0.13 (15 μL/min), 0.16 (20 μL/min), 0.21 (25 μL/min), 0.22 (30 μL/min), 0.22 (35 μL/min), 0.24 (40 μL/min), 0.22 (45 μL/min), 0.21 (50 μL/min), and 0.21 (55 μL/min), respectively. The separation performance of the device remained relatively stable across a TFR range of 35–45 μL/min (Figure 2b). The 1 μm particles were stably confined to a focused flow stream, while the 5 μm particles were arranged along the channel sidewalls under the combined effects of inertial lift forces and elastic forces. Thus, the separation performance exhibited weak sensitivity to the TFR within the specified flow rate range for the given channel length (2 cm). A TFR of 40 μL/min (sample solution: inner sheath: outer sheath= 7:28:5 μL/min) was utilized for subsequent experiments.

### 4.2. Effect of PEO Concentration on Separation Performance

To examine the effect of PEO concentration on separation performance, experiments were conducted using PEO solutions with concentrations of 0, 20, 50, 100, 200, 500, and 1000 ppm with TFR= 40 μL/min and FRR = 4:1. In each case, the particle suspensions were prepared in the corresponding PEO solutions, which were also employed as the fluids for the inner sheath flows. For instance, when a 100 ppm PEO solution was used to dilute the sample suspension, the same 100 ppm PEO solution was applied to the inner sheath inlets. To maintain flow stability and prevent backflow, phosphate-buffered saline (PBS) was introduced into the outer sheath inlet at a constant flow rate of 5 μL/min.

**Figure 3:**
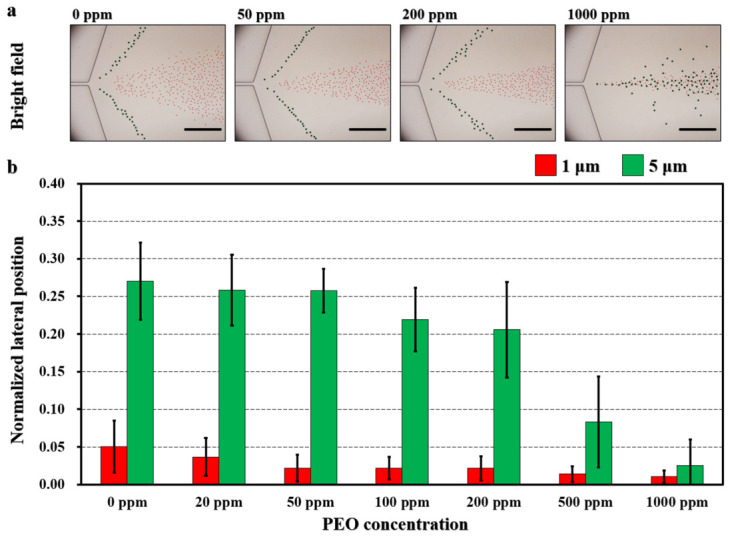
Effect of PEO concentration on the separation performance of 1 μm (red) and 5 μm (green) particles at a TFR of 40 μL/min. (**a**) Superimposed bright-field images showing particle distributions at the expansion region. Scale bar is 200 μm. (**b**) Normalized lateral positions of the particles. PEO concentrations were 0, 20, 50, 100, 200, 500, and 1000 ppm, with a fixed FRR of 4 between the inner sheath and sample flows. The PBS outer sheaths were injected at 5 μL/min. *N* = 200 for each condition.

Superimposed bright-field images showed that both 1 μm and 5 μm particles remained aligned near the channel center at the expansion region under all tested conditions (Figure 3a). As the PEO concentration increased from 0 ppm to 1000 ppm, the normalized lateral positions of the 5 μm particles decreased from 0.27 to 0.03 (Figure 3b). Similarly, the normalized lateral positions of the 1 μm particles shifted from 0.05 at 0 ppm to 0.01 at 1000 ppm. Over the examined concentration range, the differences in average normalized lateral positions between the 1 μm and 5 μm particles were 0.22 (0 ppm), 0.22 (20 ppm), 0.24 (50 ppm), 0.20 (100 ppm), 0.18 (200 ppm), 0.07 (500 ppm), and 0.01 (1000 ppm), respectively. This increasing separation distance indicates enhanced size-dependent differentiation. This behavior is attributed to the higher elastic force generated at elevated PEO concentrations, which promotes stronger particle migration toward the channel center, thereby overcoming the competing shear-gradient lift forces [36].

### 4.3. Effect of the Sheath-to-Sample FRR on Separation Performance

To evaluate the influence of the FRR between the inner sheath and sample flows on separation performance, six FRR conditions (0, 1, 2, 4, 8, and 16) were examined under a fixed TFR of 40 μL/min, while maintaining the outer PBS sheath flow at 5 μL/min. Particle suspensions were prepared in a 50 ppm PEO solution, which was also employed as the working fluid for the inner sheath streams. Lateral positions of 1 μm and 5 μm particles within the expansion region of the microchannel were subsequently recorded and analyzed.

**Figure 4:**
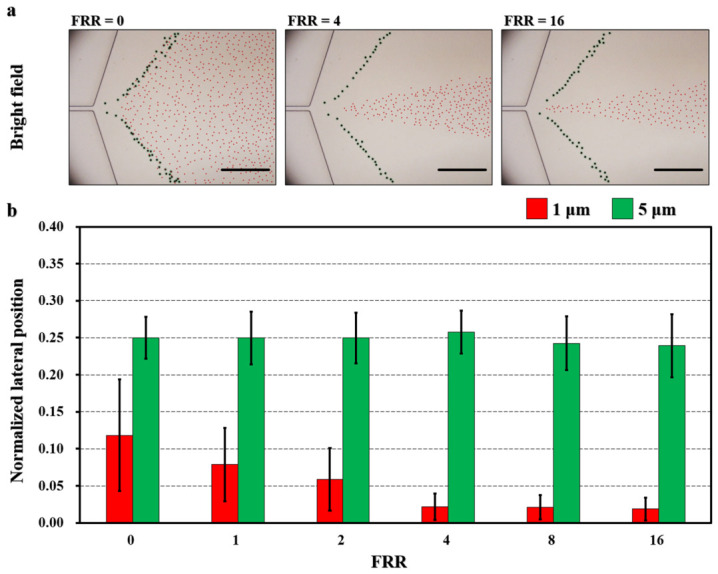
Effect of FRR on the separation performance of 1 μm (red) and 5 μm (green) particles at a TFR of 40 μL/min. (**a**) Superimposed bright-field images illustrating particle distributions within the expansion region of the microchannel. Scale bar: 200 μm. (**b**) Normalized lateral positions of the particles under FRR values of 0 (sheathless), 1, 2, 4, 8, and 16. *N* = 200 for each condition.

It was observed that, as the FRR increased, the 1 μm particles progressively focused into a narrower flow stream near the channel center, whereas the lateral positions of the 5 μm particles remained relatively stable, as shown in the superimposed bright-field images (Figure 4a). Quantitative analysis showed that the normalized lateral positions of the 1 μm particles gradually decreased with increasing FRR: 0.12 (FRR = 0), 0.08 (FRR = 1), 0.06 (FRR = 2), 0.02 (FRR = 4), 0.02 (FRR = 8), and 0.02 (FRR = 16) (Figure 4b). This trend can be attributed, on one hand, to the widening of the inner sheath flow region with increasing FRR, which hydrodynamically pinches the 1 μm particles into a narrow flow stream near the channel center. On the other hand, due to their small size, 1 μm particles experience negligible shear-gradient lift forces and are therefore more susceptible to sheath-induced pinching, resulting in progressive stream narrowing as the FRR increases. By contrast, the 5 μm particles were sufficiently large to experience significant lateral migration driven by inertial lift forces, enabling them to remain stably focused near the channel sidewalls. Consequently, their normalized lateral positions remained relatively stable, ranging from 0.26 (FRR = 4) to 0.24 (FRR = 16). Across the examined FRR range, the differences in average normalized lateral positions between the 1 μm and 5 μm particles were 0.13 (FRR = 0), 0.17 (FRR = 1), 0.19 (FRR = 2), 0.24 (FRR = 4), 0.22 (FRR = 8), and 0.22 (FRR = 16), respectively. Considering the balance between throughput and separation performance, an FRR of 4 was selected for subsequent experiments.

## 5. Separation Regulation by the Outer Sheath Flows

In this work, the separation distance between 1 and 5 μm particles at the expansion was modulated using the outer sheath flows with either varying PEO concentrations or flow rates. Based on our previous results, particle suspensions were prepared in a 50 ppm PEO solution, with the same solution employed as the working fluid for the inner sheath streams. The inner sheath flow rate and the sample flow rate were set at 28 and 7 μL/min, respectively.

### 5.1. Influence of PEO Concentration

To investigate the effect of PEO concentration on regulating the lateral separation distance between 1 μm and 5 μm particles in the expansion region, the outermost PEO sheath flow was prepared at concentrations of 0 ppm, 50 ppm, 100 ppm, 200 ppm, 500 ppm, and 1000 ppm while maintaining a constant flow rate of 5 μL/min. The lateral positions of both particle sizes at the expansion region were subsequently recorded and quantitatively analyzed.

**Figure 5:**
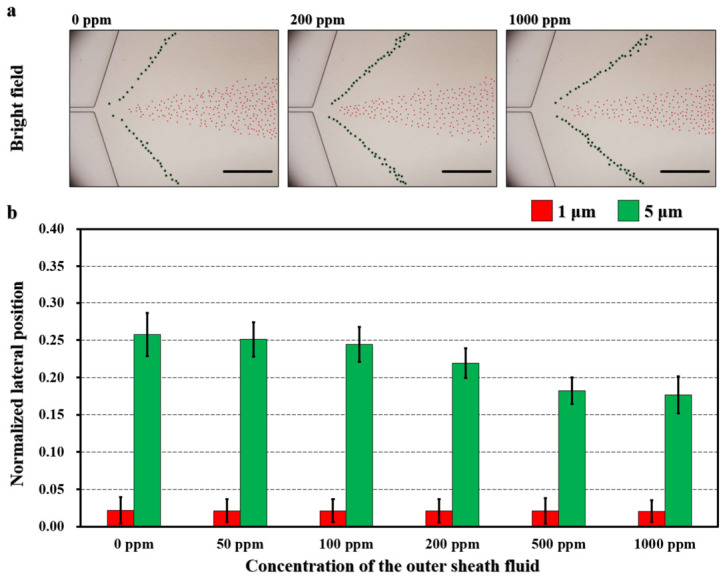
Effect of the outer sheath PEO concentration on tuning the separation distance between 1 μm (red) and 5 μm (green) particles in the expansion region of the microchannel. (**a**) Superimposed bright-field images illustrating the particle distribution under varying outer sheath PEO concentrations. Scale bar is 200 μm. (**b**) Normalized lateral particle positions. Outer sheath PEO concentrations of 0, 50, 100, 200, 500, and 1000 ppm were examined at a fixed sheath flow rate of 5 μL/min. *N* = 200 for each condition.

Superimposed bright-field images indicate that the lateral positions of 1 μm particles remain relatively stable across all PEO concentrations, whereas increasing PEO concentration progressively drives the 5 μm particles toward the channel center (Figure 5a). Quantitative analysis confirms that the standardized lateral positions of 1 μm particles are stably confined around 0.02. In contrast, the lateral positions of 5 μm particles shift significantly toward the center as the PEO concentration increases, with recorded values of 0.26 (0 ppm), 0.25 (50 ppm), 0.24 (100 ppm), 0.22 (200 ppm), 0.18 (500 ppm), and 0.18 (1000 ppm) (Figure 5b). A video was used to compare the regulatory response under 0 ppm and 1000 ppm conditions (Video S2). Correspondingly, the separation distance between 1 μm and 5 μm particles, expressed as the difference in normalized lateral positions, gradually decreases from 0.24 (0 ppm) and 0.23 (50 ppm) to 0.22 (100 ppm), 0.20 (200 ppm), 0.16 (500 ppm), and 0.16 (1000 ppm). Notably, at high PEO concentrations (e.g., 1000 ppm), the enhanced viscoelastic effects strengthen the lateral migration of 5 μm particles toward the centerline. This is likely due to the interfacial blocking effect formed between the 50 ppm and 1000 ppm streams, which restricts the lateral displacement of larger particles and promotes their confinement near the central region [20,36].

### 5.2. Effect of Outer Sheath Flow Rate (OSFR)

To investigate the influence of the OSFR on the lateral separation distance between 1 μm and 5 μm particles in the expansion region, outer sheath flows with PEO concentrations of 200 ppm and 1000 ppm were examined. For each concentration, eight flow rates ranging from 5 to 75 μL/min, in increments of 10 μL/min, were tested.

**Figure 6:**
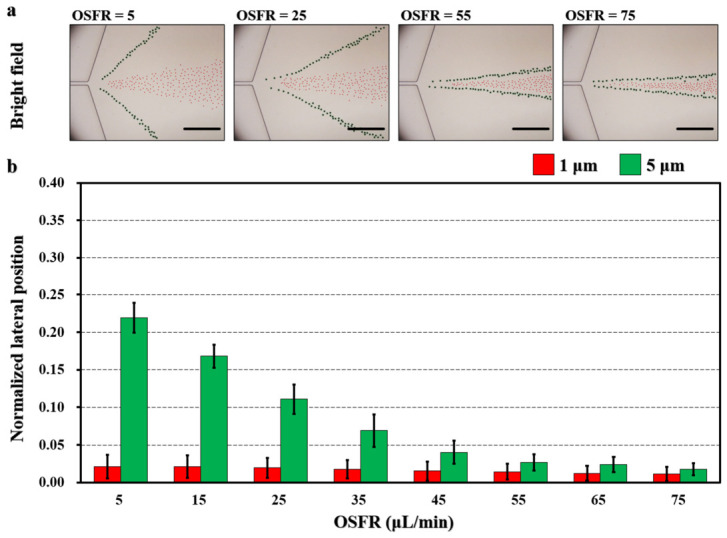
Effect of the 200 ppm outer sheath flow rate (OSFR) on the separation of 1 μm (red) and 5 μm (green) particles in the expansion region of the microchannel. (**a**) Superimposed bright-field images showing the lateral migration behavior at different OSFRs varying from 5 to 75 μL/min. Scale bar: 200 μm. (**b**) Normalized lateral positions of 1 μm and 5 μm particles. *N* = 200 for each condition.

In the 200 ppm PEO scheme, increasing the OSFR progressively drove both 1 μm and 5 μm particles toward the channel center (Figure 6a). As the OSFR increased from 5 to 75 μL/min, the normalized lateral positions of the 1 μm particles shifted from 0.02 to 0.01, while those of the 5 μm particles changed from 0.22 to 0.02. Correspondingly, the separation distances between the two particle sizes were 0.20 (5 μL/min), 0.15 (15 μL/min), 0.09 (25 μL/min), 0.05 (35 μL/min), 0.03 (45 μL/min), 0.01 (55 μL/min), 0.01 (65 μL/min), and 0.01 (75 μL/min), respectively (Figure 6b). A video was provided to compare the regulatory response under 5 μL/min and 25 μL/min conditions (Video S3). These results demonstrate that real-time modulation of the separation distance between 1 μm and 5 μm particles in the expansion region can be effectively achieved by tuning the OSFR. The increase in OSFR introduced more fluid between the channel sidewalls and the particles, thereby pushing the particles toward the channel center. In this work, the equilibrium positions of the particles are more sensitive to the OSFR. In the 1000 ppm PEO scheme, the separation distances observed at flow rates of 5, 15, 25, 35, 45, 55, 65, and 75 μL/min were 0.16, 0.07, 0.04, 0.03, 0.01, 0.01, 0.01, and 0.00, respectively (Figure S2). Compared to the 1000 ppm outer sheath, the 200 ppm sheath exhibited a stronger capability to regulate the separation distance. This is likely because the interface formed between the 50 ppm inner sheath and the 200 ppm outer sheath was less resistant, allowing 5 μm particles to be more influenced by the outer flow field.

Additionally, scenarios of 0 ppm, 50 ppm, 100 ppm, and 500 ppm were also investigated, and the adjustable ranges for 5 μm particles across all these conditions were compared (Table S2). Through bright-field observation of the motion trajectories of 5 μm particles, it was found that under 0 ppm, 50 ppm, and 100 ppm conditions, as the flow rate increased, the particle trajectories exhibited significant curvature (Figures S3–S5). This is likely due to the relatively weak interfacial resistance provided by the outer sheath flow within this concentration range. In contrast, under 200 ppm, 500 ppm, and 1000 ppm conditions, the outer sheath flow was able to provide sufficient interfacial resistance, allowing the migration trajectories of 5 μm particles to remain relatively straight (Figure 6, Figures S2 and S6).

Specifically, using TFR as the evaluation criterion, the viscoelastic microfluidic chip in this study (with an optimal separation flow rate of 40 μL/min and a maximum tunable flow rate of 110 μL/min) exhibited a higher throughput compared to existing DLD [28] and PFF [26] technologies (0.05–1.33 μL/min). In contrast, compared to existing viscoelastic microfluidics (50–160 μL/min) [16], although the throughput of this chip was potentially lower, its greater number of outlets provided superior system tunability. Moreover, through systematic parameter adjustment and multiple particle model experiments as well as biological sample experiments, this chip was validated to have good reproducibility.

## 6. Separation of *E. coli* and *C. vulgaris*

To investigate the separation capability of the microfluidic device for biological samples, a mixed sample of *E. coli* and *C. vulgaris* with PEO and PBS, along with PEO and PBS sheath fluids, was injected into the microchannel. The sample flow rate was set at 7 μL/min (50 ppm), the inner sheath flow rate at 28 μL/min (50 ppm), and the OSFRs were set to 5 μL/min and 25 μL/min (200 ppm), respectively.

**Figure 7:**
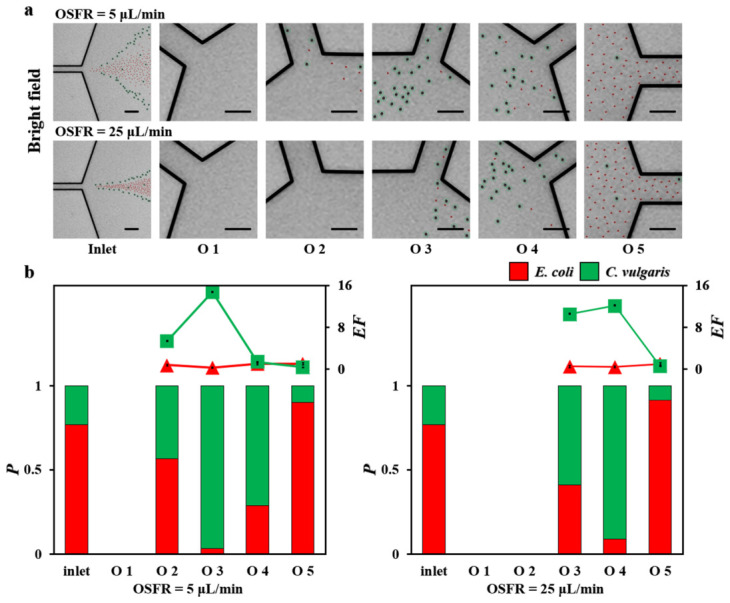
Separation of *E. coli* and *C. vulgaris* in the channel expansion region under different outer sheath flow rates (OSFRs). (**a**) Bright-field images showing the distribution of *E. coli* and *C. vulgaris* at the inlet and each outlet, with *E. coli* and *C. vulgaris* marked by red and green circles, respectively. Scale bar: 40 μm. (**b**) Analysis of the *purity* (*P*) and *enrichment factor* (*EF*) of *E. coli* and *C. vulgaris* at each outlet under different OSFRs. *N* = 200 for each condition.

The bright-field distribution of *E. coli* and *C. vulgaris* at the inlet and each outlet under different flow rate conditions was presented (Figure 7a). When the OSFR was 5 μL/min, *C. vulgaris* mainly flowed out through outlet 3, while *E. coli* mainly flowed out through outlet 5. Under this condition, the collection amounts of both *E. coli* and *C. vulgaris* at outlet 1 were extremely low. When the OSFR was increased to 25 μL/min, *C. vulgaris* mainly flowed out through outlet 4, whereas *E. coli* still mainly flowed out through outlet 5. In this case, the collection amounts of both cell types at outlets 1 and 2 were low. Two quantitative parameters were used to evaluate the separation efficiency, namely *purity* (*P*) and *enrichment factor* (*EF*). Here, *P* was defined as the ratio of the number of specific particles to the total number of particles, while the *EF* was defined as the ratio of the particle proportion at a certain outlet to that at the inlet.

The *P* and *EF* of the two cell types at each outlet under different flow rate conditions were presented (Figure 7b). When the OSFR was 5 μL/min, *C. vulgaris* reached its highest purity at outlet 3 (*p* = 0.97), with a corresponding *EF* of 14.8. Outlet 1 was excluded from the analysis because the number of collected cells of both species was extremely low. When the OSFR was increased to 25 μL/min, *C. vulgaris* achieved its highest purity at outlet 4 (*p* = 0.91), with an *EF* of 12.2, while outlets 1 and 2 were excluded due to insufficient collection of both cell types. Under both flow rate conditions, *E. coli* reached its highest purity at outlet 5, with a purity of 0.90 at an OSFR of 5 μL/min and 0.92 at 25 μL/min. At the higher flow rate, the purity of *E. coli* at outlet 5 showed a slight increase, but the improvement was limited, mainly attributed to the increased number of *E. coli* entering this outlet, along with a concurrent increase in the influx of *C. vulgaris*. In addition, the distribution of *E. coli* within the microchannel was concentrated near the centerline region. With a channel depth of 50 μm, *E. coli* cells located at different depths may not be effectively observed. It should be noted that the inherent size non-uniformity of *E. coli* and *C. vulgaris* may reduce the purity of the separation of the chip. However, the established microfluidic platform holds potential for processing complex samples with size disparities, e.g., the separation of red blood cells and platelets [37] or the separation of yeast cells and bacteria [38], through the manipulation of fluid rheologies. However, this platform was limited by the relatively complex preparation of the viscoelastic fluid and potential interference of polymer molecular chains with downstream detection and analysis under certain conditions. Furthermore, the established microfluidic platform is expected to be incapable of separating nanoscale particles, as their extremely small size renders the inertial lift force insufficient to induce effective lateral migration, thereby leading to poor separation performance. Future optimization of the channel design hopes to enable the separation of nanoparticles using viscoelastic flows.

## 7. Conclusions

This study successfully designed and implemented a novel microfluidic platform featuring two pairs of symmetric viscoelastic sheath-flow channels, enabling simultaneous size-dependent particle separation and dynamic regulation of separation performance within a simple straight-channel architecture. The results demonstrate that in a PEO solution at a specific concentration (50 ppm), with the assistance of the first pair of viscoelastic sheath flows, 1 μm and 5 μm particles exhibited significantly distinct migration behaviors under the combined action of elastic and inertial forces, thereby enabling size-based separation. By adjusting the viscoelastic properties and flow rates of the PEO solution in the second pair of sheath-flow channels, the spatial spacing between the two particle streams could be regulated in real time. Further experiments revealed that higher-concentration PEO solutions (500 and 1000 ppm) exerted stronger deflection effects on the particle streams, while lower concentrations (50, 100, and 200 ppm) offered greater flexibility in fine-tuning the separation outcome. The work further demonstrated tunable separation performance for *E. coli* and *C. vulgaris*, highlighting its applicability in biological particle processing. This research provides a microfluidic platform with combined separation and regulation functions for biomedical applications such as real-time detection, cell separation, and targeted drug delivery, holding significant scientific importance and practical value.

## Data Availability

The data are available upon reasonable request.

## Supplementary Materials

The following supporting information can be downloaded at: https://www.mdpi.com/article/10.3390/bios16050273/s1, Table S1: Reynolds number (*Re*), Weissenberg number (*Wi*), and elasticity number (*El*) corresponding to viscoelastic solutions at different concentrations. The total flow rate is 40 μL/min (sample solution: inner sheath flow: outer sheath flow = 7:28:5 μL/min); Table S2: Adjustable range of the average normalized lateral position of 5 μm particles for outer sheath solutions at different concentrations. This range is defined as the average normalized lateral position at the minimum outer sheath flow rate (OSFR) (5 μL/min) minus that at the maximum OSFR (75 μL/min). The sample solution and inner sheath flow rates are set at a ratio of 7:28 μL/min, both with a concentration of 50 ppm; Figure S1: Numerical simulation of flow velocity distribution for illustrating the lateral migration of 1 μm (pink) and 5 μm (green) particles in a 50 ppm viscoelastic solution (total flow rate: 40 μL/min; outer sheath fluid: 0 ppm PBS solution at 5 μL/min; inner sheath fluid: 50 ppm PEO solution at 28 μL/min; sample fluid: 50 ppm PEO solution at 7 μL/min). $F_{L}$ and $F_{E}$ mean the inertial lift force and the elastic force, respectively. $F_{LS}$ and $F_{LW}$ indicate the shear-gradient lift force and wall-induced lift force, respectively; Figure S2: Effect of the 1000 ppm outer sheath flow rate (OSFR) on the separation of 1 μm (red) and 5 μm (green) particles in the expansion region of the microchannel. (a) Superimposed bright-field images showing the lateral migration behavior at different OSFRs varying from 5 to 75 μL/min. Scale bar: 200 μm. (b) Normalized lateral positions of 1 μm and 5 μm particles. N = 200 for each condition; Figure S3: Effect of the 0 ppm OSFR on the separation of 1 μm (red) and 5 μm (green) particles in the expansion region of the microchannel. (a) Superimposed bright-field images showing the lateral migration behavior at different OSFRs varying from 5 to 75 μL/min. Scale bar: 200 μm. (b) Normalized lateral positions of 1 μm and 5 μm particles. N = 200 for each condition; Figure S4: Effect of the 50 ppm OSFR on the separation of 1 μm (red) and 5 μm (green) particles in the expansion region of the microchannel. (a) Superimposed bright-field images showing the lateral migration behavior at different OSFRs varying from 5 to 75 μL/min. Scale bar: 200 μm. (b) Normalized lateral positions of 1 μm and 5 μm particles. N = 200 for each condition; Figure S5: Effect of the 100 ppm OSFR on the separation of 1 μm (red) and 5 μm (green) particles in the expansion region of the microchannel. (a) Superimposed bright-field images showing the lateral migration behavior at different OSFRs varying from 5 to 75 μL/min. Scale bar: 200 μm. (b) Normalized lateral positions of 1 μm and 5 μm particles. N = 200 for each condition; Figure S6: Effect of the 500 ppm OSFR on the separation of 1 μm (red) and 5 μm (green) particles in the expansion region of the microchannel. (a) Superimposed bright-field images showing the lateral migration behavior at different OSFRs varying from 5 to 75 μL/min. Scale bar: 200 μm. (b) Normalized lateral positions of 1 μm and 5 μm particles. N = 200 for each condition; Video S1: A video demonstrating the separation performance at a flow rate of 40 μL/min in the expansion region was provided (Video S1); Video S2: A video was used to compare the regulatory response under 0 ppm and 1000 ppm conditions (Video S2); Video S3: A video was provided to compare the regulatory response under 5 μL/min and 25 μL/min conditions (Video S3).

## Abbreviations

The following abbreviations are used in this manuscript:

PEO	Polyethylene Oxide
DNA	Deoxyribonucleic Acid
XG	Xanthan Gum
PVP	Polyvinylpyrrolidone
*E. coli*	*Escherichia coli*
PFF	Pinched Flow Fractionation
DLD	Deterministic Lateral Displacement
PDMS	Polydimethylsiloxane
*C. vulgaris*	*Chlorella vulgaris*
*Re*	Reynolds Number
*El*	Elasticity Number
*Wi*	Weissenberg Number
PBS	Phosphate-Buffered Saline
TFR	Total Flow Rate
FRR	Flow Rate Ratio
OSFR	Outer Sheath Flow Rate
*P*	*P**urity*
*EF*	*Enrichment Factor*
