# Supplementary material for: Tunable Particle Separation in a Straight Microchannel via Symmetrical Viscoelastic Sheath Flows
Source: Biosensors (Basel). 2026 May 8;16(5):273. doi: 10.3390/bios16050273 (PMC13204369; doi:10.3390/bios16050273)
Supplement: Supplementary file 1 [file biosensors-16-00273-s001.zip › Supporting information-Final Proofreading.pdf]

Supporting Information

Tunable Particle Separation in a Straight Microchannel via Symmetrical Viscoelastic Sheath Flows

Tianyuan Zhou [1], Qi Cui [1], Guizhong Tian [1], Ping Liu [2], Yoichiroh Hosokawa [3], Yaxiaer Yalikun [3], Shilun Feng [4,*], Tianlong Zhang [1,*]

[1] College of Mechanical Engineering, Jiangsu University of Science and Technology, Zhenjiang 212100, China

[2] School of Mechanical and Electrical Engineering, Suqian University, Suqian 223800, China

[3] Medilux Research Center, Nara Institute of Science and Technology, Nara, 630-0192, Japan

[4] State Key Laboratory of Transducer Technology, Shanghai Institute of Microsystem and Information Technology, Chinese Academy of Sciences, Shanghai, 200050, China

* Correspondence: shilun.feng@mail.sim.ac.cn(S.F.); tianlong.zhang23@just.edu.cn(T.Z.)

Table S1. Reynolds number (*Re*), Weissenberg number (*Wi*), and elasticity number (*El*) corresponding to viscoelastic solutions at different concentrations. The total flow rate is 40 μL/min (sample solution: inner sheath flow: outer sheath flow = 7:28:5 μL/min).

PEO concentration (c, ppm)	0	20	50	100	200	500	1000
Reynolds number (Re)	16.70	20.00	19.76	19.37	18.64	16.73	14.30
Weissenberg number (Wi)	NA	2.57	4.66	7.31	11.46	20.80	32.64
Elasticity number (El)	NA	0.13	0.24	0.38	0.62	1.24	2.28

Table S2. Adjustable range of the average normalized lateral position of 5 μm particles for outer sheath solutions at different concentrations. This range is defined as the average normalized lateral position at the minimum outer sheath flow rate (OSFR) (5 μL/min) minus that at the maximum OSFR (75 μL/min). The sample solution and inner sheath flow rates are set at a ratio of 7:28 μL/min, both with a concentration of 50 ppm.

PEO concentration (c, ppm)	0	50	100	200	500	1000
Tunable range of 5 μm particles	0.16	0.20	0.21	0.20	0.17	0.16

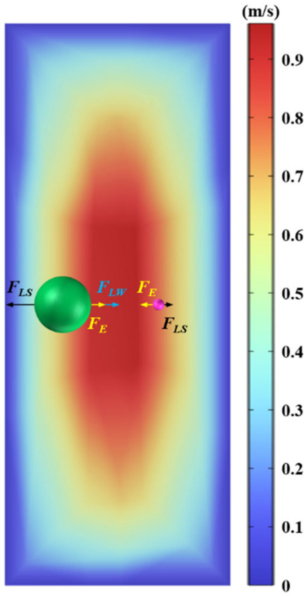

Figure S1. Numerical simulation of flow velocity distribution for illustrating the lateral migration of 1 μm (pink) and 5 μm (green) particles in a 50 ppm viscoelastic solution (total flow rate: 40 μL/min; outer sheath fluid: 0 ppm PBS solution at 5 μL/min; inner sheath fluid: 50 ppm PEO solution at 28 μL/min; sample fluid: 50 ppm PEO solution at 7 μL/min). F_L and F_E mean the inertial lift force and the elastic force, respectively. F_{LS} and F_{LW} indicate the shear-gradient lift force and wall-induced lift force,

respectively.

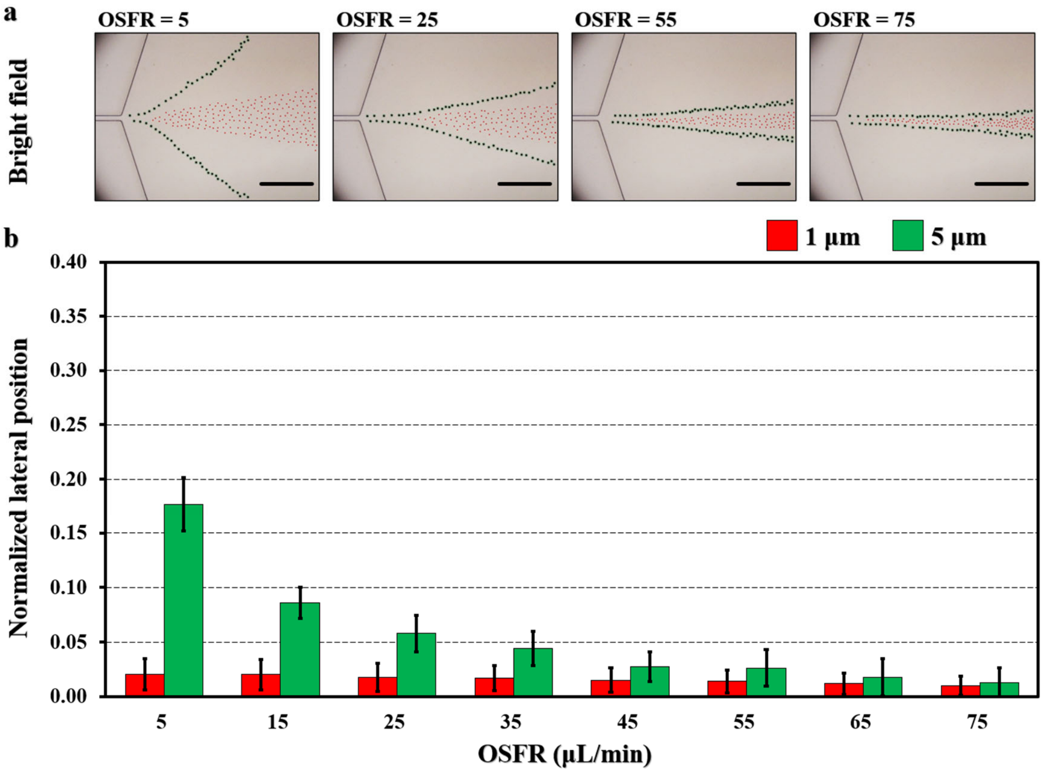

Figure S2. Effect of the 1000 ppm outer sheath flow rate (OSFR) on the separation of 1 µm (red) and 5 µm (green) particles in the expansion region of the microchannel. (**a**) Superimposed bright-field images showing the lateral migration behavior at different OSFRs varying from 5 to 75 µL/min. Scale bar: 200 µm. (**b**) Normalized lateral positions of 1 µm and 5 µm particles. $N = 200$ for each condition.

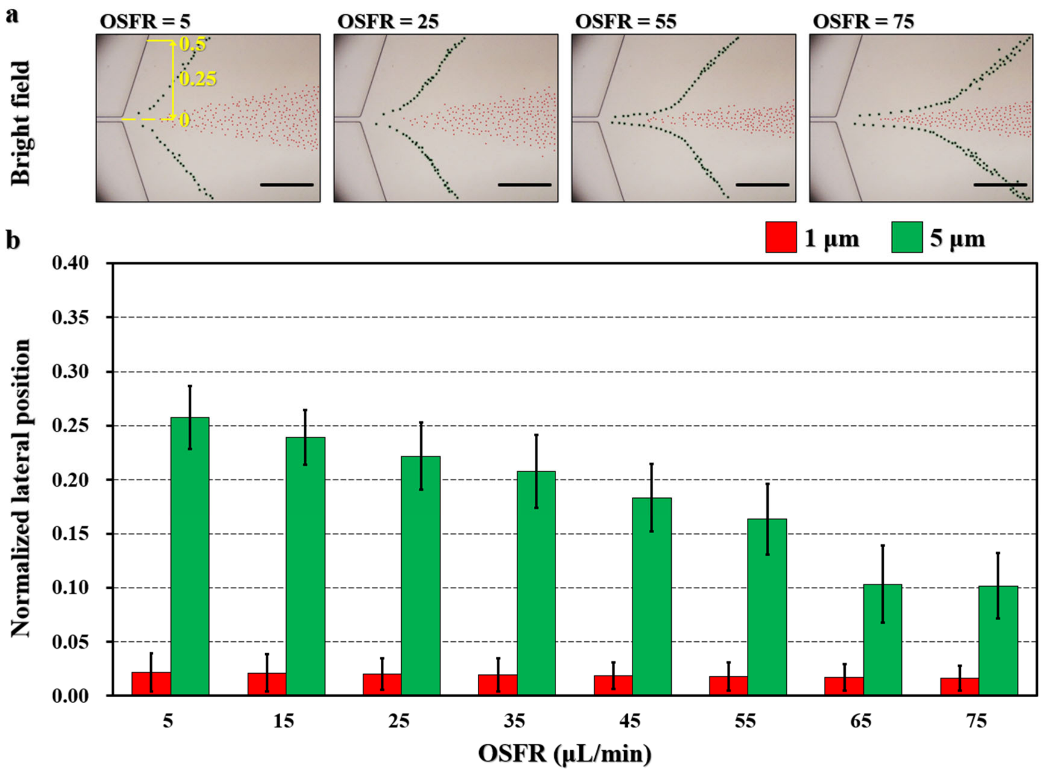

Figure S3. Effect of the 0 ppm OSFR on the separation of 1 μm (red) and 5 μm (green) particles in the expansion region of the microchannel. (**a**) Superimposed bright-field images showing the lateral migration behavior at different OSFRs varying from 5 to 75 μL/min. Scale bar: 200 μm. (**b**) Normalized lateral positions of 1 μm and 5 μm particles. N = 200 for each condition.

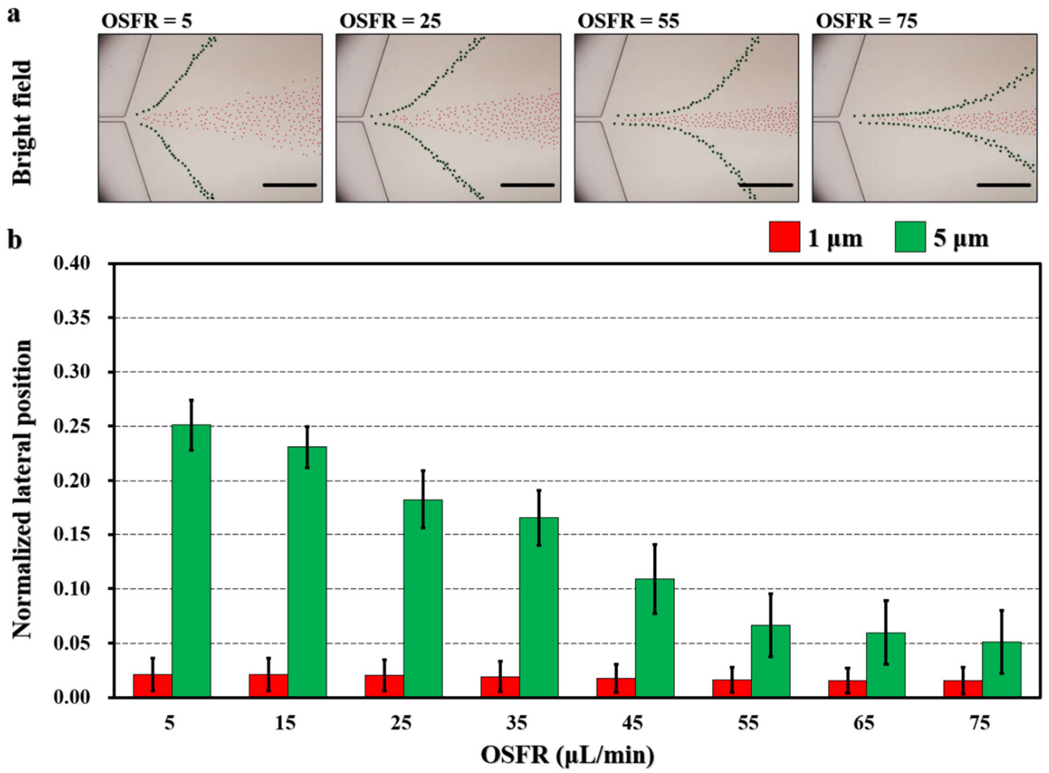

Figure S4. Effect of the 50 ppm OSFR on the separation of 1 μm (red) and 5 μm (green) particles in the expansion region of the microchannel. (**a**) Superimposed bright-field images showing the lateral migration behavior at different OSFRs varying from 5 to 75 μL/min. Scale bar: 200 μm. (**b**) Normalized lateral positions of 1 μm and 5 μm particles. $N = 200$ for each condition.

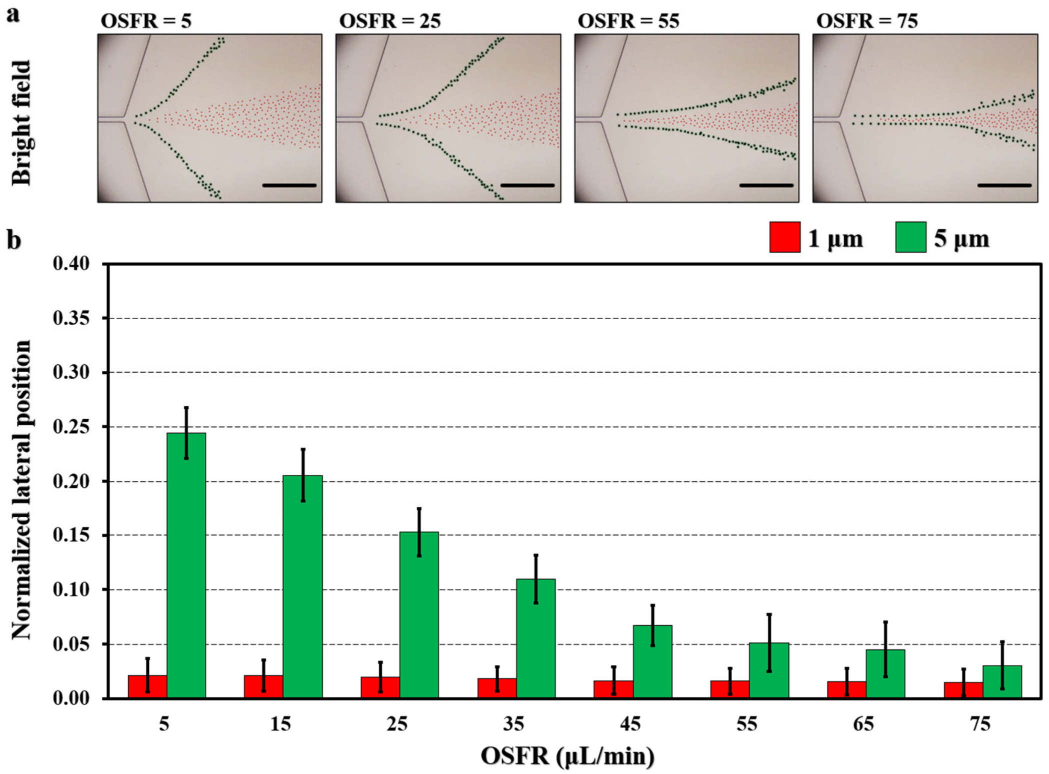

Figure S5. Effect of the 100 ppm OSFR on the separation of 1 µm (red) and 5 µm (green) particles in the expansion region of the microchannel. (**a**) Superimposed bright-field images showing the lateral migration behavior at different OSFRs varying from 5 to 75 µL/min. Scale bar: 200 µm. (**b**) Normalized lateral positions of 1 µm and 5 µm particles. $N = 200$ for each condition.

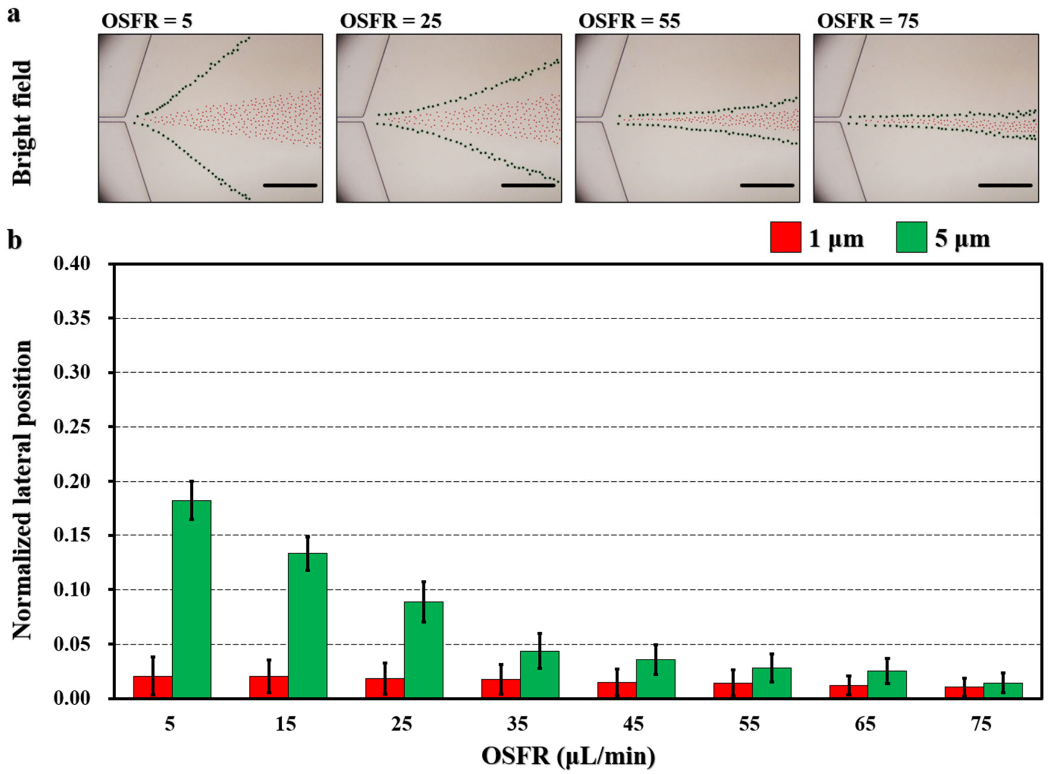

Figure S6. Effect of the 500 ppm OSFR on the separation of 1 μm (red) and 5 μm (green) particles in the expansion region of the microchannel. (**a**) Superimposed bright-field images showing the lateral migration behavior at different OSFRs varying from 5 to 75 μL/min. Scale bar: 200 μm. (**b**) Normalized lateral positions of 1 μm and 5 μm particles. $N = 200$ for each condition.
